# BVD seroprevalence in the Irish cattle population as the national BVD programme progresses toward eradication

**DOI:** 10.1186/s12917-022-03318-0

**Published:** 2022-06-01

**Authors:** Damien Barrett, AnneMarie Clarke, Kate O’Keeffe, Padraig Kellegher, John Comerford, Elizabeth Lane, Andrew W. Byrne

**Affiliations:** 1 Department of Agriculture, Food and the Marine, One Health One Welfare Scientific Support Team, Agriculture House, Kildare St, Dublin 2, Ireland; 2 School of Veterinary Medicine, University College Dublin, Belfield, Dublin 4, Ireland; 3 Cork Blood Testing Laboratory, Dept of Agriculture, Food and the Marine, Model Farm Rd, Cork, Ireland; 4 Department of Agriculture, Food and the Marine, Veterinary Public Health Inspection Service, Agriculture House, Kildare St., Dublin 2, Ireland; 5 Department of Agriculture, Food and the Marine, Animal Health Division, Agriculture House, Kildare St, Dublin 2, Ireland

**Keywords:** Bovine viral diarrhoea virus, Serology tests, Ireland, Disease surveillance, Disease eradication

## Abstract

**Background:**

Bovine Viral Diarrhoea Virus (BVDV) infection remains endemic in many countries worldwide. Ireland, in common with several other European counties, commenced an BVDV eradication programme in the last decade, Managing eradication programmes requires careful monitoring of diseases prevalence and understanding factors associated with disease exposure to ensure eradication programmes remain evidence based and tailored to the evolving epidemiological situation.

**Methods:**

In this study, we explore the seroprevalence of BVDV exposure over a four-year period (2017 to 2020) in Ireland from a cohort of animals (*n* = 6,449) under 30 months of age sampled at slaughter, who were born subsequent to the commencement of a compulsory national eradication programme. Temporal trends and risk factor analysis were undertaken using multilevel logit regression models.

**Results:**

There was a declining temporal trend in seroprevalence over the sample years of the study, and risk varied at both county- and herd-levels. The unadjusted marginal animal-level seroprevalence reduced from 9.1% in 2017 (95%; CI: 7.2—10.9) to 3.9% in 2020 (95%; CI: 3.2—4.6). The final model suggested that seropositivity in study cattle was strongly related with the presence of a PI animal in the herd during the year of the animal’s birth, and to a lesser extent the status of the herd from which the animal was slaughtered. The risk of seroconversion increased significantly with increasing size of the herd of slaughter, in females relative to males, and in dairy relative to suckler herds.

**Conclusions:**

This study has shown that the BVDV serostatus of cattle at slaughter is correlated to the BVD infection history of the herd into which the animal was born and the herd from which it was slaughtered. Herd location, increased herd size and dairy production were associated with increased probability of serconversion. These findings will be used to inform the targeting of surveillance strategies once BVDV freedom has been achieved.

**Supplementary Information:**

The online version contains supplementary material available at 10.1186/s12917-022-03318-0.

## Background

Bovine viral diarrhoea virus (BVDV) is a member of the genus Pestivirus, family Flaviviridae, and is an economically important pathogen of cattle worldwide, present at high prevalence in many countries around the world [1]. Scandinavian countries have successfully eradicated this disease and several countries in Europe have national control programmes operating, including, Belgium, Germany, Austria and Ireland [2]. In Ireland, in the absence of an effective control scheme, losses due to BVDV were estimated at €102illion annually [3]. As a consequence, a voluntary industry-led eradication programme commenced in 2012 [4], progressing to a compulsory national programme, supported by legislation, from 1st January 2013 [5] Considerable progress has been made since the programme commenced where the BVDV animal prevalence has decreased from 0.67% in 2013 to 0.03% in 2021 [6], and the prevalence of BVDV positive herds has reduced from 11.3% in 2013 to 0.52% of herds in 2021.

On foot of this progress, Ireland is approaching the requirements of BVDV freedom under the Animal Health Law (AHL; EU Regulation 2020/690). When BVDV freedom is achieved, the testing regime will likely change from testing of individual animals for evidence of virus to serological surveillance, with a focus moving from providing a status for each individual animal and herd to providing an assurance of national freedom. Under the AHL, 99.8% of herds comprising 99.9% of animals in the country must be free of BVDV. It is envisaged that BVDV freedom will be demonstrated using a combination of bulk milk serology for dairy cattle and abattoir based serological surveillance for beef cattle [7].

In other countries, bulk milk serology, young stock screens and abattoir serological surveillance have been used to demonstrate exposure to BVDV [8]. While some research has been conducted on the use of bulk milk serology, young stock serology, and abattoir surveillance as tools to assess exposure to BVD virus [9, 10], there is limited data available on the seroprevalence of BVDV exposure among Irish cattle born since the commencement of the national BVDV eradication programme in 2013 and the potential use of abattoir-based serology as a BVDV surveillance tool in the Irish cattle population.

A previous Irish study that surveyed a subset of herds sampled for the national Brucellosis programme in 2009 found in excess of 98% of herds contained animals seropositive for BVDV [11]. A subsequent on-farm survey of 161 suckler herds found a 100% herd seroprevalence for BVDV and mean within-herd level prevalence of 77.7% (median 85.2%) [12]. In that study, BVDV within-herd seroprevalence was positively associated with increasing herd size, increased herd mortality, reduced herd productivity as measured by calves produced per cow per year and co-infection with neosporosis.

The objectives of this study were two-fold: the first to determine the prevalence of BVDV seroconversion among a group of cattle slaughtered at less than 30 months of age over a four-year period from 2017 to 2020, and the second to identify any herd-level risk factors associated with exposure to BVDV to inform the potential targeting of serological surveillance.

## Material & methods

### Serological surveillance

Three sets of data were collated over a four-year period in order to estimate the BVDV exposure in Irish cattle, using sera collected for routine serological surveillance purposes. The animals were selected randomly from under 30 month of age cattle going for slaughter. A minimum sample size of 1,013 for each annual survey was calculated to determine true prevalence based on a design prevalence of 10%, test sensitivity and specificity of 95% and 99% respectively, with a 95% confidence (https://epitools.ausvet.com.au).

Serum was collected from cattle under 30 months of age at the time of their slaughter in 26 abattoirs across Ireland, in January 2017, April 2018 and July 2020. These animals were considered reflective of the population born since the commencement of the BVD eradication scheme in 2013. The samples in each of the three groups were tested at the Cork Blood Testing Laboratory. They were tested for BVD antibody using the IDEXX BVDV/MD/BVD p80 Protein Antibody Kits, according to the manufacturers’ instructions.

### Ethical statement on sample collection:

Serum was obtained from blood samples which were collected for routine national animal disease surveillance purposes. From an ethical perspective, the material collected and used as part of this study was outside the scope of Directive 2010/63. All samples were collected post mortem and as such sample collection did not come under the scope of any welfare guidelines.

### Data management & analysis

The animal identity and test result data were entered on to spreadsheets (Microsoft Excel). Movement and registration data for these animals were downloaded from the Department of Agriculture, Food and Marine’s Animal Identification and Movement System (AIMS) database and incorporated into the spreadsheets. Animal Health Ireland (https://animalhealthireland.ie/) provided the data on BVDV herd infection status. Data manipulation and statistical analysis was carried out using Stata 16 (StataCorp LP, Texas, USA). A descriptive statistical analysis of the data was initially conducted, followed by a univariable and multivariable regression analyses.

### Independent variables

Descriptive information on the independent variables assessed is presented in Table 1. Temporal, animal-level, and herd-level variables were explored as potential candidate variables of interest. Data were collected on three different years (2017, 2018, 2020) over a four-year period (2017 to 2020). Herd type was taken as the broad categorisation made within the AIMS database. Where necessary variables were transformed or categorised (for example, by splitting into quartiles) to improve model fit. Birth and last herd size were measured at two time points during respective years and compared in competing univariable models – year quartile (q) 1 average and q4 average. There was > 94% correlation between the two within-year metrics, with a slightly better support for the Q4 metric based on Akaike’s Information Criteria (AIC). There was a 24% correlation between birth and last herd size metrics, therefore both variables were added into candidate multivariable models with some risk of variance inflation. The movement of animals was measured in two ways – firstly whether an animal moved at any point during its life, that is whether the animal’s last herd was different to its birth herd. A second movement metric was the number of movements an animal made, as recorded in the AIMs database, for the year prior to sample. The relationship with the outcome was assessed as a binary variable (yes/no moved within previous year) and as a categorical variable. Herds during the year of the animal’s birth were categorised as BVDV positive if one or more animals were disclosed with either a BVD PCR or ELISA test positive in that year, using data derived from the national BVD eradication database from 2014 until 2020.

**Table 1:** Descriptive statistics and unadjusted univariable associations from logistic regression models between animal-level BVD serology test status and temporal, animal-, and herd-level independent variables

*Independent variable*	BVD antibody -ve (%)	BVD antibody + ve (%)	Total	OR	Lower 95%CI	Upper 95%CI	*P*-Value
TEMPORAL							
* Year*							
* 2017*	1071 (91.15)	104 (8.85)	1175	1.000			referent
* 2018*	2286 (94.42)	135 (5.58)	2421	0.608	0.466	0.793	< 0.001
* 2020*	2742 (96.11)	111 (3.89)	2853	0.417	0.316	0.550	< 0.001
ANIMAL LEVEL							
* Age – months (quartiles)*							
* 10–23*	1500 (93.05)	112 (6.95)	1,612	1.000			referent
* 24–25*	1284 (95.11)	66 (4.89)	1,350	0.688	0.019	0.503	0.942
* 26–27*	1365 (93.88)	89 (6.12)	1,454	0.873	0.356	0.655	1.164
* 28–30*	1950 (95.92)	89 (4.08)	2,033	0.570	0.000	0.426	0.763
* Breed*							
* Aberdeen Angus*	1,116 (94.98)	59 (5.02)	1175	1.000			referent
* Charolais*	1,124 (95.09)	58 (4.91)	1182	0.976	0.673	1.416	0.898
* Friesian*	1,170 (93.23)	85 (6.77)	1255	1.374	0.976	1.935	0.069
* Hereford*	892 (94.99)	47 (5.01)	939	0.997	0.673	1.477	0.987
* Limousin*	1,137 (94.51)	66 (5.49)	1203	1.098	0.765	1.575	0.612
* Other*	660 (94.96)	35 (5.04)	695	1.003	0.653	1.541	0.989
* Sex*							
* Male*	3513 (95.44)	168 (4.56)	3681	0.679	0.548	0.843	< 0.001
* Female*	2,586 (93.42)	182 (6.58)	2768	1.000			referent
* Movement (different last herd to birth herd)*							
* No*	1733 (94.49)	101 (6.51)	1,834	1.000			referent
* Yes*	4366 (94.6)	249 (5.43)	4,615	0.979	0.771	1.241	0.858
* Moved during previous year*							
* No*	1677 (94.59)	96 (5.41)	1,773	1.000			referent
* Yes*	4422 (94.57)	254 (5.43)	4,676	1.003	0.735	1.370	0.983
HERD LEVEL							
* Herd Type (last)*							
* Beef*	337 (92.84)	26 (7.16)	363	1.160	0.761	1.770	0.489
* Dairy*	3,264 (93.77)	217 (6.23)	3481	1.000			referent
* Suckler*	2,192 (96.31)	84 (3.69)	2276	0.576	0.446	0.746	< 0.001
* Other*	306 (93.01)	23 (6.99)	329	1.131	0.724	1.765	0.589
* Birth Herd size (quartiles)*							
< *60 (mean: 33.7)*	1515 (95.83)	66 (4.17)	1,581				referent
* 60–119 (mean:88.9)*	1548 (94.39)	92 (5.61)	1,640	1.364	0.987	1.886	0.06
* 120–201 (mean: 157.4)*	1536 (95.17)	78 (4.83)	1,614	1.166	0.834	1.630	0.37
* 202–1574 (mean: 352.7)*	1500 (92.94)	114 (7.06)	1,614	1.745	1.278	2.382	< 0.001
* Last Herd size (quartiles)*							
< *52 (mean: 28.9)*	1537 (96.00)	64 (4.00)	1,601				referent
* 53–109 (mean: 80.6)*	1543 (95.66)	70 (4.34)	1,613	1.089	0.771	1.540	0.628
* 110–225 (mean: 160.3)*	1519 (93.88)	99 (6.12)	1,618	1.565	1.134	2.160	0.006
* 226–3755 (mean: 451.2)*	1500 (92.76)	117 (7.24)	1,617	1.873	1.370	2.561	< 0.001
* PI Birth herd year status*							
* Absent*	5890 (95.52)	276 (4.48)	6166	1.000			referent
* Present*	209 (73.85)	74 (26.15)	283	7.556	5.339	10.694	< 0.001
* PI Last herd year status*							
* Absent*	6003 (94.92)	321 (5.08)	6,324	1.000			referent
* Present*	96 (76.8)	29 (23.2)	125	5.649	3.674	8.686	< 0.001

### Statistical approach

Throughout the outcome was a binary variable, representing the seropositivity status of sampled animals, modelled using a logit distribution. Unconditional unadjusted associations were explored using logistic regression analyses.

A fixed effect multivariable logit regression model was built including all putative risk factors associated with the outcome at univariable level (*p* < 0.2; Table 1). Standard errors were adjusted for clustering within birth herds. A backwards elimination strategy (both manual and semi-automated using the ‘stepwise’ commend) was used to identify the most parsimonious model. Competing final models were compared using Akaike’s Information Criteria (AIC).

Additional multivariable models were built to take into account the hierarchical structure within the dataset, with animals clustering within herds and herds within counties. Therefore, final multilevel hierarchical logit models were developed to assess the relationship between putative risk factors for serology positivity for cattle sampled in Ireland, with random effects for both herd and county using the melogit suite of commands in Stata 16. Comparisons were made between multi-level model which fitted the data better than a nested fixed effect model using likelihood ratio tests. Furthermore, a comparison of models (fixed effect, 2-level random effect (herd id > animal), 2-level random effect (county > animal), 3-level random effect (county > herd id > animal)) using Akaike’s information criteria (AIC) was undertaken to assess which was the most supported model structure. Model performance was assessed using Area Under the ROC curve (AUC), and value that can range from 0 to 1, with higher values indicating superior discriminatory ability. Generally, models with AUC > 0.7 are considered “adequate”, while models with AUC > 0.9 are considered “excellent” or “outstanding”. For the random effects model, the ROC was calculated using both the fixed effects only and with the inclusion of the random effects. Given the predictions from the model, using a fixed cutpoint equal to the proportion of the sample test positive, apparent sensitivity and specificity was calculated.

## Results

Overall, there were 350 test positive serology tests within the dataset of 6,449 < 30-month-old animals tested, providing an animal level seroprevalence of 5.43%. There were 75 suspect cases (1.16%), which were considered negative in this study.

**Fig. 1:**
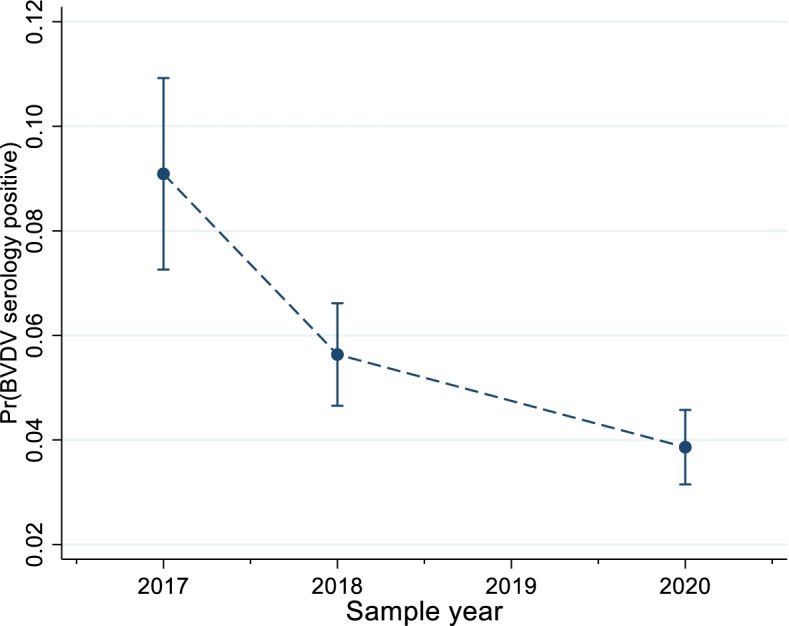
Mean marginal probability of cattle testing positive for BVDV exposure via a serology test during three samples years from 2017–2020 in Ireland

There was a significant declining trend in serology positivity over the three years of the survey (Odds ratio (OR): 0.76 (95% Confidence Interval (CI): 0.70—0.84); *P* < 0.001; Fig. 1).

Modelling year as a categorical factor demonstrated that much of this decline occurred during 2017–2018. The marginal predicted probability of being serology positive in 2017 was 0.09 (95% CI: 0.07—0.11), declining to 0.06 (95% CI: 0.05—0.07) in 2018, and to 0.04 in 2020 (95% CI: 0.03—0.05).

### Univariable analysis

Univariable associations between animal-level BVDV serology test status and temporal, animal-level, and herd-level independent variables are presented in Table 1. Unconditional significant associations were found between BVDV serology status and year, sex, herd type, herd size of birth and last herds, and BVD PI status for last and birth herds (Table 1). Details of the univariable associations are presented in Supplementary Material.

### Multivariable models

The final best supported fixed-effect multivariable model contained sample year, sex, last herd herd-size, herd type of last herd, and BVD PI status of last and birth herds, respectively, and county. There was no evidence of a significant lack of fit to the data (Hosmer–Lemeshow: χ^2^(df:8) = 13.9; Prob > χ^2^ = 0.09). The model had an area under the ROC curve (AUC) of 0.73. At a cut-point of 0.543 (mean proportion positive in sample), the model exhibited apparent sensitivity of 62.9% and specificity of 72.5%. Because of the hierarchical structure within the dataset, we focus on the final multivariable hierarchical random effects model (Table 2).

**Table 2:** Final mixed effects logistical regression model of BVDV seroprevalence

Independent variables	Odds Ratio	Std. Err	z	P > z	Lower 95%CI	Upper 95%CI
BVD birth herd status in year of birth (Referent: Negative)						
* BVD positive herd in year of birth*	20.889	8.033	7.900	< 0.001	9.831	44.385
BVD last herd status in year of birth (Referent: Negative)						
* Herd of slaughter BVD virus positive*	6.565	3.066	4.030	< 0.001	2.629	16.395
Herd type (referent: dairy)						
* Beef*	1.504	0.567	1.080	0.280	0.718	3.149
* Other*	0.798	0.352	-0.510	0.609	0.337	1.892
* Suckler*	0.387	0.095	-3.860	< 0.001	0.239	0.626
Sex (Referent: Female)						
* Male*	0.538	0.102	-3.270	0.001	0.370	0.780
Log(last herd size)	1.376	0.128	3.440	0.001	1.148	1.651
Sample year (linear predictor)	0.779	0.065	-2.980	0.003	0.661	0.918
Constant	0.004	0.003	-7.700	< 0.001	0.001	0.015
Random effects						
county						
Variance	0.217	0.137			0.063	0.750
county > herd						
Variance	6.816	1.753			4.117	11.283

**Fig. 2:**
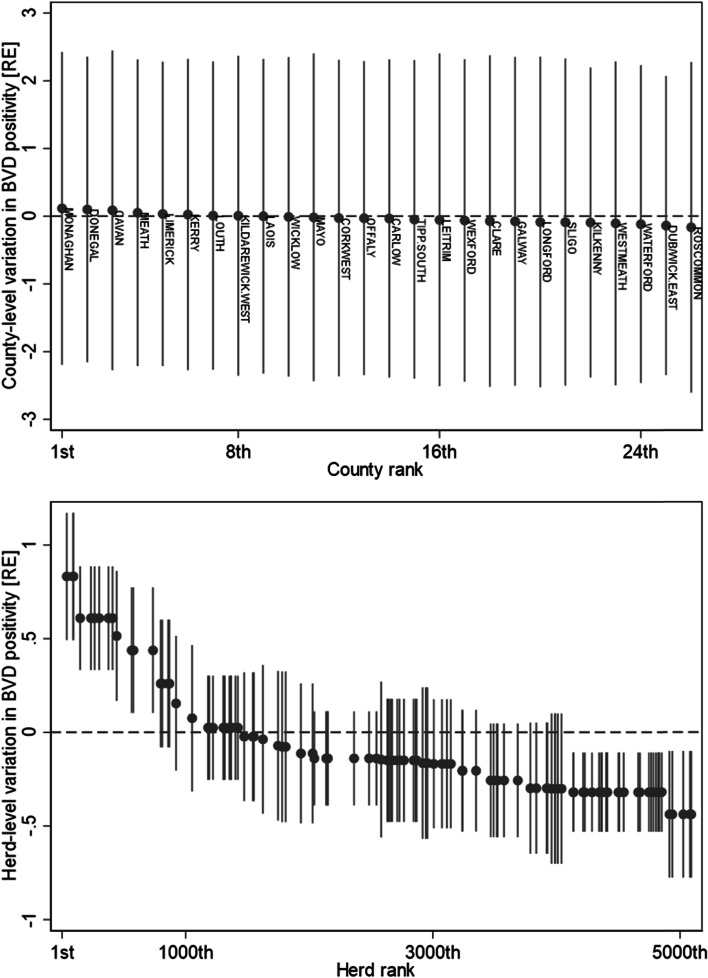
Ladder plot county-level (top panel) and herd-level* (bottom panel) random effect variance in BVD serology risk from a hierarchical model. * Note, only a 2% random sample of herds are presented in the figure so individual herds could be visualised

A likelihood ratio test suggested that the multi-level model fitted the data better than a nested fixed effect model, therefore the hierarchical structure should be accounted for (df = 2; χ^2^ = 115.29; *p* < 0.001). Furthermore, a comparison of models (fixed effect, 2-level random effect (herd id > animal), 2-level random effect (county > animal), 3-level random effect (county > herd id > animal)) using Akaike’s information criteria (AIC) suggested that the 3-level random effects model was the most supported model. The final hierarchical model exhibited a ROC value of 69.8% for the marginal model integrating out the random effects; including the random effects, the ROC value was 99.6%, highlighting the importance of clustering of infection within the dataset. The variance in the serology risk across the herd-level and county-level random effects are presented in Figs. 2. Monaghan, Donegal, Cavan, and Meath were highest rank, while Dublin/East-Wicklow, Waterford, Westmeath and Roscommon were the lowest rank, but within county variance was substantial (Fig. 2). The ladder plot for variance at herd-level suggested that there was more variation in the mean risk across herds (Fig. 2). This was due to there being 5,122 unique herds in the dataset, with the average of 1.3 animals per herd (range: 1 – 15). In contrast, the average county had 248 associated animal records (range 29–1103). The intra-class correlation coefficient (ICC) for the county-level was 0.02 (95% CI: 0.01–0.07), while the ICC was 0.68 for the herd-within-county level (95% CI: 0.56–0.78). This indicates that serology positivity is only slightly correlated within the same county. The ICC values indicate that county and herd random effects together compose ~ 68% of the total residual variance, controlling for the fixed effects in the model.

**Fig. 3:**
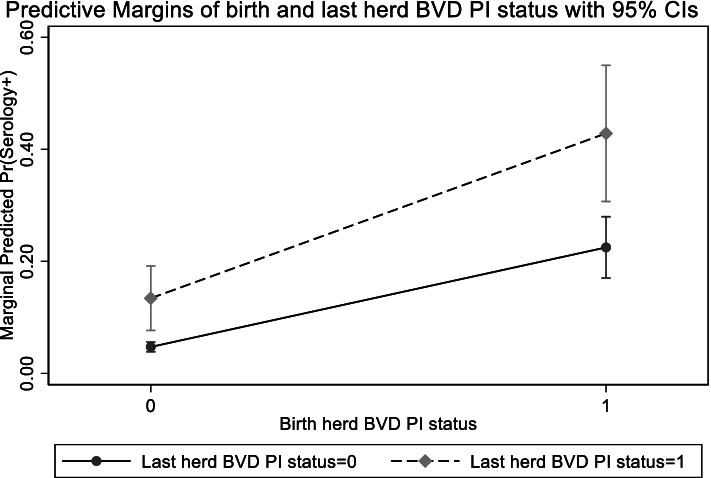
Predicted marginal probability of animal’s being serology positive in relation to their birth and last herd of residence PI BVD year status

**Fig. 4:**
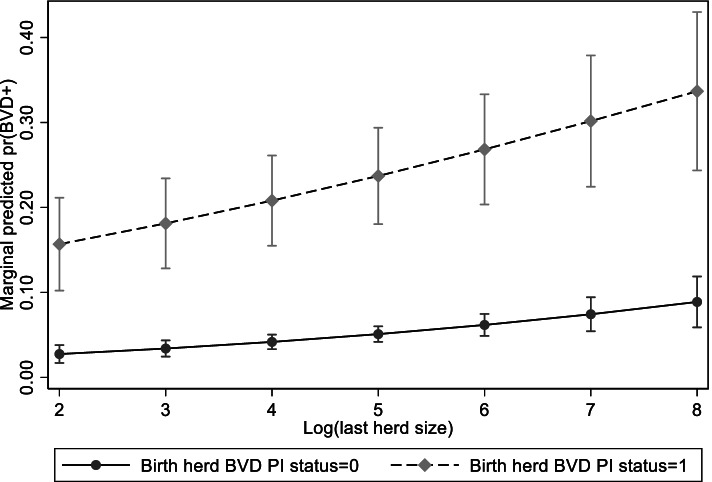
Predicted marginal probability of animal’s being serology positive in relation to herd size and depending on birth herd PI BVD year status

The fixed effects part of the model suggested that the outcome was strongly affected by the status of their birth herd during the year of birth (OR: 20.89; 95%CI: 9.83—44.39), and to a lesser extent the herd from which they resided before slaughter and sampling (“last herd” OR: 6.56; 95%CI: 2.63–16.39). The marginal predicted probability varied from 0.05 for animals from negative birth and last herds, through to 0.48 for animals that resided in herds with PI animals during both their birth and sample years (see Fig. 3). The hierarchical model suggested that serology positivity was associated with females relative to males and being sampled from a dairy herd than a suckler (Table 2). There was an association with increasing herd size (Fig. 4), such that animals from very small herds (exp (2) ≈ 7 animals) mean marginal predicted probability of seropositivity was 0.03, rising to 0.10 for animals from the largest herds (exp (8) ≈ 2,981 animals).

The temporal declining odds of being serology positive was modelled as a linear predictor, with 22% (95% CI: 8%—34%) reduction in odds with each increasing year.

## Discussion

This is the first study to describe the seroprevalence of BVDV among cattle known to be born since the initiation of the national BVD eradication in Ireland. The BVD seroprevalences among under 30-month old cattle slaughtered in 2017, 2018 and 2020 were 9.1%, 5.6% and 3.9% respectively, which is a marked improvement on previous seroprevalence studies [11, 12]. Our analysis suggests that there was a significant year-on-year reduction in the seroprevalence of BVDV over the course of the study, mirroring the significant achievements thus far reported for the national programme. Our results demonstrate how important the infectious status of the birth herd and to a lesser extent the herd from which the animal was slaughtered, in predicting adult animals seropositivity status at slaughter.

The rate of BVD seroconversion demonstrated among under 30-month old cattle at slaughter over the course of the study is substantially less than the mean prevalence of 77% previously reported in Irish suckler cows [11], which indicates a marked reduction in the exposure to BVDV in the Irish cattle population. These findings are consistent with the reduction in the prevalence of BVDV in the Irish cattle population since the commencement of the national BVD eradication programme, where the animal-level prevalence has decreased from 0.67% in 2013 to 0.03% in 20,210 [6], the prevalence of BVDV positive herds has fallen from 11.3% in 2013 to 0.527% of herds in 2021 [6].

When BVDV freedom is achieved, it is likely that the current practice of individually testing all calves will cease and serological surveillance will be used to provide evidence for the proof of freedom, similar to the processes currently used in Sweden [8] and it will be especially important to target surveillance towards risk herds in the earlier stages of disease so that any circulating BVDV is promptly identified and stamped out to prevent onward transmission within the cattle population and undo the disease eradication effort.

A positive BVDV infection status of the herd of birth, in the year of the animal’s birth was the strongest predictor of animal being BVDV seropositive at slaughter, with an OR of almost 21 in the final hierarchical model. It is reassuring that there is such a strong association between the serostatus of the animal at slaughter and the infection status of its birth herd in the year the animal was born. The disclosure of cattle persistently infected with BVDV is usually associated with the circulation of BVDV in the herd in the previous breeding season [13]. Animals may also have come in contact with BVDV in their second year, i.e. the years subsequent to their births. We detected associations with lagged herd BVD status during the year prior and post birth, albeit not as strong as the association with the herd infection status in the year of birth of the animal (data not shown). This applies to all herds in which the animal resided in during its life, and it was noted that there was also an association in the final multivariable model between the serostatus of the animals at slaughter and the BVD infection status of the herd from which the animal was slaughtered.

It was noted that approximately 71% of animals were slaughtered out of the herds other than the herds in which they were born, which is consistent with relatively high level of cattle movement in Ireland [14]. It was noteworthy however that most animals had one or fewer movements in the year preceding their slaughter. However, a small number had four or more movements. The disclosure of a BVD antibody positive animal in a herd warrants further investigation in all the herds in which it resided and this forms the basis of BVD surveillance in those countries which are free of BVDV [8]. However, the lack of an association with movement history would suggest the focus of such surveillance should, in the first instance, be on the birth and slaughter herds of seropositive animals.

Though the herd-level association with dairy enterprises has been equivocal across studies [15–17], over the course of the eradication programme in Ireland significantly more dairy herds and large herds have been affected by BVDV [5, 18]. During the present study there was significantly less seroconversion among suckler bred cattle, relative to animals sampled from dairy herds. Herd size as risk factor for disease is well documented [19] and this may in part account for the increased risk of seroconversion among calves born in dairy herds, as they tend to be larger than suckler herds [18]. In the current study, both dairy and larger herd size contributed to elevated risk additively. These findings suggest there may be value in targeting surveillance activities at dairy herds and larger herds in general, as have been demonstrated in Germany [20].

County was also retained in the final fixed effect model, which indicates a spatial element in the risk of exposure to BVDV. The multilevel random effects model ranked counties in terms of risk, which controlling for the herd random effect and the fixed effects. It was noteworthy that the three counties with the highest risk of BVDV exposure from this model were in Donegal, Monaghan, and Cavan which border Northern Ireland. While Northern Ireland has a BVDV eradication programme in place [21], it is not as far advanced as the Irish programme, and there are close trade and cultural relationships which cross the border. A high prevalence of BVDV and increased spatial BVDV risk in two borders areas have been documented in Northern Ireland [17, 19].

Under 30-month-old cattle were selected for these surveys as they were born since the commencement of the Irish BVDV programme and were the largest suitable age cohort available for sampling. If any of these animals had seroconverted, it would indicate circulation of BVDV in their herds of residence since their birth. Surveying youngstock more than six months of age, when maternal antibodies would have waned, may be considered preferable as it would be more likely to be reflective of the current situation within the herd, as there may be a lag between seroconversion and detection of seropositive animals at slaughter. However, there is no readily available means to access serum from that youngstock age cohort on farms. The youngest available age group in an abattoir in Ireland would have been under 16-month old bulls, but the numbers of cattle and herds associated with this production system is relatively small and unlikely would not be representative of the wider population.

BVDV vaccines will continue to be available for use to Irish farmers until BVD freedom is achieved, and it is possible that seroconversion could come about as a result of vaccination rather than exposure to BVDV. However, it is unlikely that cattle destined for slaughter would be vaccinated for BVDV, as it is used primarily for breeding female cattle to prevent the development of persistently infected carriers. Additionally, since the commencement of the BVDV eradication programme there has been a marked decline in the use of BVDV vaccines in Ireland. We cannot completely rule out the possibility that seroconversion could have occurred due to vaccination rather than exposure to natural infection. However, the use of BVDV vaccination in the age cohort and enterprise type is negligible, so therefore it is unlikely vaccination contributed to the seroconversion detected in any meaningful way.

## Limitations

Firstly, the data were generated as part of other surveillance activities, and therefore were not explicitly designed solely for the purposes of this paper. Secondly, we had an interrupted time-series, as there was a lack of resources available to undertake the survey during 2019. The study was retrospective and observational, and therefore we always have to be cognisant of the limitations regarding causal inference for such study designs. The sampling was based on a convenience simple random sample, with samplers advised to take one sample per herd batch. The involvement of all 26 abattoirs slaughtering under 30 month old cattle ensures good spatial representation. This type of surveillance system will be of particular value for proof of freedom once BVDV freedom has been achieved, but is of lesser value as a case detection tool due to the delay between detection of antibodies and the exposure to the persistently infected animal which led to the development of antibodies.

## Conclusion and implications

The seroprevalences described in this current study are substantially less than those previously documented in previous Irish studies. Whatsmore, there are year on year decreases in seroprevalence, paralleling the progress being made in the national eradication programme. However, the level of progress being documented todate is not sufficient to met the requirements for proof of freedom outlined in the Animal Health Law Del. Reg. (EU) 2020/689).

This study has shown that the BVD serostatus of cattle at slaughter is well correlated to the BVD infection history of the herd into which the animal was born and the herd from which it was slaughtered. The identification of herd location, increased herd size and dairy production as risk factors associate will increased probability of serconversion as a result of BVDV exposure due to the circulation of the virus. This information will be used to inform the targeting of surveillance strategies once BVDV freedom has been achieved.

## Supplementary Information

**Additional file 1.** 

## Data Availability

The datasets generated and/or analysed during the current study are not publicly available due to data protection legislation, but may be made available from the corresponding author on reasonable request.

## Abbreviations

AIC: Akaike’s Information Criteria
AIMS: Animal Identity and Movement System
AUC: Area Under the ROC curve
BVDV: Bovine viral diarrhoea virus
CI: Confidence Interval
ICC: Intra-class correlation coefficient
OR: Odds Ratio
SD: Standard Deviation
