# Supplementary material for: BVD seroprevalence in the Irish cattle population as the national BVD programme progresses toward eradication
Source: BMC Vet Res. 2022 Jun 1;18:210. doi: 10.1186/s12917-022-03318-0 (PMC9158290; doi:10.1186/s12917-022-03318-0)
Supplement: Supplementary file 1 — Additional file 1. [file 12917_2022_3318_MOESM1_ESM.docx]

**Supplementary Material**

**Univariable associations**

Most animals only made 0 or 1 movement (25^th^ percentile=0; 50^th^ percentile 1; mean: 1.18), however a very small number of animals made four or more movements (99^th^ percentile; max = 8); 71.6 % of animals had a different slaughter herd than their birth herd. However, there were no associations with BVDV serology status and movement metrics (including change in herd over lifetime, binary recent move, and number of moves over the previous year). Furthermore, there was no association with BVDV serology status and breed or age.

There was a higher proportion of females positive (182 of 2,586; 6.58%) than males (168 of 3,513; 4.56%; OR male: 0.68; 95% CI: 0.55 - 0.84). Animals from suckler herds had an odds ratio of 0.58 (p < 0.001; 95% CI: 0.45 - 0.75) relative to dairy herds for being BVD seropositive. There was no significant difference in risk across other herd types (i.e. dairy vs beef vs other, all pairwise tests p > 0.4). There was large variance in the herd-size variables (Figure S1), with the average size being 158.53 and the median of 120 animals, and a maximum herd size of 1,574 for birth herds. There was a trend towards animals having higher odds of serocoverting in larger herds (either birth or last herd; Figure S2) – for example, the odds ratio for animals sampled in herds in quartile 4 of the herd size (mean: 451.24; SD: 333.15; n=1,617) distribution relative to quartile 1 (mean: 28.86; SD:14.12; n= 1,601), was 1.873 (95% CI: 1.37-2.56). Herd-size was log_n_ transformed to improve the model fit. The odds ratios for each log unit increase in herd size was 1.198 (95% CI: 1.06-1.34) and 1.29 (95% CI: 1.17-1.43) for birth and last herds, respectively.

There was significant variation in spatial risk across counties (Figure S3; Supplementary material Table S1). The highest predicted risk, not accounting for other risk factors, was for animals born in herds in Donegal, Monaghan, Meath, and Kerry, with mean probability of being BVDV positive > 0.089. The four lowest risk herds were Carlow, Longford, Mayo, Roscommon, with the mean probability of animals born in these counties being BVDV positive <0.029.

The largest effect sizes [14] were found between BVD status and herd BVD status for either the location of the animal’s birth or the last herd the animal resided when sampled. The odds ratio from an unadjusted logistic model was 7.56 (95% CI: 5.34-10.69; P<0.001) for being born into a herd with a PI disclosed during that year. Fitting a (subject specific) random effects model to the data, controlling for multiple observations per birth herd, provides an odds ratio of 20.99 (95% CI: 9.62 - 45.76), reflecting the between-herd variance in the dataset [15]. The odds ratio from an unadjusted logistic regression for serology positivity status and last herd BVD year status was 5.65 (95% CI: 3.67-8.67; p<0.001). However, the subject specific odds ratio from a random effects model was 6.293 (95% CI: 2.97-13.34), indicating less between-herd variation.

**Figure S1:** Distribution of the birth herd sizes of study animals. Right hand side graph is raw herd size; left hand side is the log-transformed herd size.

**Figure S2:** Univariable relationship between serology positivity and the log_n_-transformed herd size of birth herds. Blue bars represent 95% CI; green line is a locally weighted regression (LOWESS) fit to the data.

**

**Fig. S3:** Marginal predicted probability of study animals from an unadjusted logistic regression model being serologically test positive for BVDV exposure in Ireland 2017-2020.

**Table S1:** Unadjusted univariable associations between animal level BVD serology test positive status and the counties from which the animals last resided. Bold parameters indicate counties with the highest % serology positive observations.

| **County** | **Neg.** | **Pos.** | **County total** | **% positive** | **OR** | **Lower 95%CI** | **Upper 95%CI** | **P-Value** |
| --- | --- | --- | --- | --- | --- | --- | --- | --- |
| CARLOW | 106 | 3 | 109 | 2.75% | 1 |  |  | Referant |
| CAVAN | 178 | 15 | 193 | 7.77% | 2.978 | 0.842 | 10.525 | 0.090 |
| CLARE | 231 | 7 | 238 | 2.94% | 1.071 | 0.272 | 4.222 | 0.922 |
| CORKWEST | 1,049 | 54 | 1,103 | 4.90% | 1.819 | 0.559 | 5.917 | 0.320 |
| **DONEGAL** | **150** | **19** | **169** | **11.24%** | **4.476** | **1.292** | **15.509** | **0.018** |
| DUBLIN/WICKLOW EAST | 27 | 2 | 29 | 6.90% | 2.617 | 0.416 | 16.454 | 0.305 |
| GALWAY | 321 | 11 | 332 | 3.31% | 1.211 | 0.332 | 4.422 | 0.772 |
| **KERRY** | **327** | **32** | **359** | **8.91%** | **3.458** | **1.038** | **11.521** | **0.043** |
| KILDARE/WICKLOW WEST | 85 | 7 | 92 | 7.61% | 2.910 | 0.730 | 11.593 | 0.130 |
| KILKENNY | 410 | 21 | 431 | 4.87% | 1.810 | 0.530 | 6.182 | 0.344 |
| LAOIS | 212 | 12 | 224 | 5.36% | 2.000 | 0.552 | 7.240 | 0.291 |
| LEITRIM | 68 | 2 | 70 | 2.86% | 1.039 | 0.169 | 6.381 | 0.967 |
| LIMERICK | 423 | 34 | 457 | 7.44% | 2.840 | 0.856 | 9.424 | 0.088 |
| LONGFORD | 70 | 2 | 72 | 2.78% | 1.010 | 0.164 | 6.196 | 0.992 |
| LOUTH | 83 | 6 | 89 | 6.74% | 2.554 | 0.620 | 10.518 | 0.194 |
| MAYO | 276 | 8 | 284 | 2.82% | 1.024 | 0.267 | 3.934 | 0.972 |
| **MEATH** | **172** | **17** | **189** | **8.99%** | **3.492** | **1.000** | **12.201** | **0.050** |
| **MONAGHAN** | **139** | **15** | **154** | **9.74%** | **3.813** | **1.076** | **13.511** | **0.038** |
| OFFALY | 153 | 10 | 163 | 6.13% | 2.309 | 0.621 | 8.591 | 0.212 |
| ROSCOMMON | 134 | 2 | 136 | 1.47% | 0.527 | 0.087 | 3.214 | 0.488 |
| SLIGO | 100 | 4 | 104 | 3.85% | 1.413 | 0.309 | 6.473 | 0.656 |
| TIPPERARY SOUTH | 604 | 34 | 638 | 5.33% | 1.989 | 0.600 | 6.593 | 0.261 |
| WATERFORD | 278 | 10 | 288 | 3.47% | 1.271 | 0.343 | 4.708 | 0.720 |
| WESTMEATH | 137 | 7 | 144 | 4.86% | 1.805 | 0.456 | 7.148 | 0.400 |
| WEXFORD | 288 | 12 | 300 | 4.00% | 1.472 | 0.407 | 5.319 | 0.555 |
| WICKLOW | 78 | 4 | 82 | 4.88% | 1.812 | 0.394 | 8.328 | 0.445 |


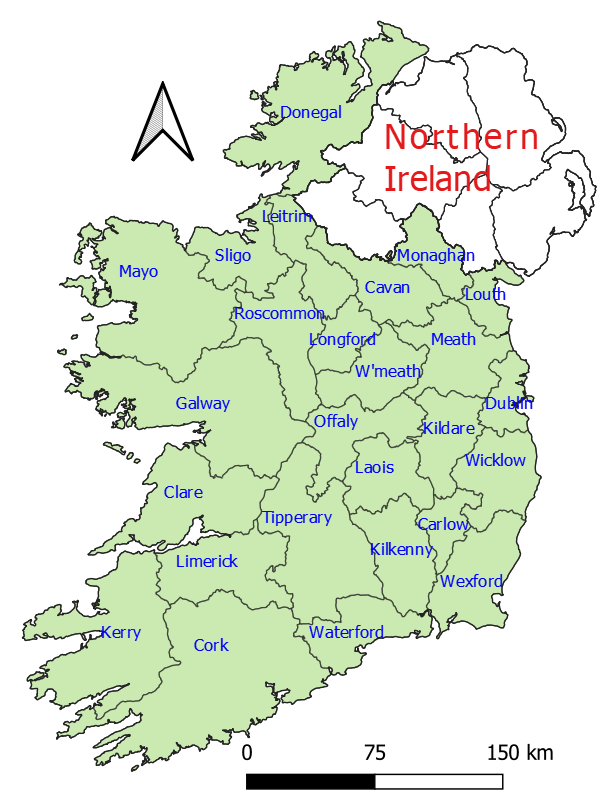


Figure X; County map of Ireland and Northern Ireland. Green polygons are counties within Ireland. White polygons are the six counties of Northern Ireland (UK).
